# 12 years of experience in intrauterine testicular torsion management

**DOI:** 10.1007/s00383-025-06093-9

**Published:** 2025-06-23

**Authors:** Olga Devrim Ayvaz, Sabri Cansaran, Ayşenur Celayir, Zeynep Eylul Erol

**Affiliations:** 1 Department of Pediatric Surgery, University of Health Sciences Turkey, Istanbul Zeynep Kamil Maternity and Children’s Diseases Health Training and Research Center, Dr. Fahri Atabey Cd., Üsküdar, 34668 Istanbul, Turkey; 2 Faculty of Medicine Kayışdağı, Yeditepe University, İnönü Mahallesi, Kayışdağı Cd., Ataşehir, 34755 Istanbul, Turkey

**Keywords:** Intrauterine testicular torsion, Management, Neonate

## Abstract

**Introduction:**

Intrauterine testicular torsion is a rare pathology and there is no consensus on how to treat and follow-up these patients. This study aims to evaluate the surgical indications, findings, testicular viability rate, and management approaches of intrauterine testicular torsion cases in our center over a 12-year period.

**Methods:**

17 patients diagnosed with intrauterine testicular torsion and operated on between January 2010 and 2023 were included in the study. The patients’ age at birth, mode of birth, weight, maternal age, genetic diagnosis, presence of other anomalies, complaints upon presentation, physical examination findings, laboratory results, preoperative ultrasound and Doppler imaging findings, surgery date, surgery weight, torsion side, surgery method and findings, torsion type (intravaginal, extravaginal), degree of torsion, length of hospital stay, complications, pathology results, testicular status in postoperative follow-up and ultrasonography and Doppler findings were examined.

**Results:**

The median age of the cases was one day. Orchiectomy was performed in fourteen cases (82.4%), while three cases (17.6%) were managed with detorsion and orchiopexy. Prophylactic contralateral orchiopexy was performed in four (23.5%) cases, and undescended testicle surgery was performed in one (5.9%) case. None of the testes managed with detorsion and orchiopexy remained viable during follow-up.

**Conclusion:**

The etiology, risk factors, and optimal management approach of intrauterine testicular torsion are not fully elucidated. Based on the current literature, the salvage rates are close to zero in our study as well as many others despite the efforts to promptly diagnose and operate intrauterine torsion cases. Creating management algorithms based on the data gathered from multiple centers over the years can yield better outcomes for this devastating pathology.

## Introduction

Testicular torsion is the rotation of the testis around the axis of the spermatic cord resulting in ischemia [1]. Testicular torsion that occurs in the first month of life is referred to as perinatal testicular torsion (PTT), which can be further subclassified as prenatal—or intrauterine—and postnatal torsion [2]. The estimated incidence of PTT is 6.1/100,000 live births [1].

Intrauterine and extrauterine torsion differs in terms of their timing, clinical presentation, and salvage rates. While intrauterine testicular torsion (ITT) presents immediately after birth often with a painless, swollen and discolored scrotum, or can be incidentally diagnosed in utero, extrauterine testicular torsion (ETT) occurs later on and presents with acute pain, swelling and redness. ITT is reported to be virtually non-salvageable whereas ETT has a better chance of viability if operated urgently. But is ITT actually a lost cause or are higher salvage rates possible with early recognition and intervention? This study aims to evaluate the clinical characteristic, imaging results, torsion type, management strategies and testicular viability rates of the children who were operated on with the preliminary diagnosis of ITT in our clinic over a 12-year period, in order to contribute to the literature on the optimal diagnosis and management of ITT cases.

## Methods

This case series analysis included patients diagnosed with ITT who were hospitalized and operated on in the pediatric surgery clinic and patients who were not diagnosed with ITT but underwent emergency surgery with a preliminary diagnosis of testicular torsion based on postnatal physical examination findings and/or postnatal ultrasonography and Doppler findings between January 2010 and 2023. The cases whose scrotal examination was completely normal at birth but had findings supporting the development of testicular torsion in the postnatal period were not included in the study.

The patients’ age at birth, mode of birth, weight, maternal age, genetic diagnosis, and presence of other anomalies were investigated. Complaints, presence of prenatal diagnosis, physical examination findings, laboratory results, preoperative ultrasound and Doppler imaging findings, surgery date, surgery weight, side (left, right, bilateral), surgery method, presence of additional surgeries in the same session, surgery findings, torsion type (intravaginal, extravaginal), degree of torsion, length of hospital stay, complications, pathology results, testicular status in postoperative follow-up and ultrasonography and Doppler findings were examined. Descriptive analysis of the data was performed using the statistical analysis program SPSS 20.0 (IBM Corp., Armonk, NY, USA).

## Results

The study included 17 newborns who were diagnosed with ITT and urgently operated. The median age of the cases was one day [2 SD2.2 days (min: 1 day, max: 9 days)]. Twelve of the patients (70.6%) were one day old. Nine (52.9%) cases were born in our hospital, and eight (47.1%) were born in external centers. Seven (41.2%) were born by cesarean section, seven (41.2%) were born by normal spontaneous vaginal delivery, and the mode of birth of three cases was not recorded in their files. The mean birth weight of the cases was 3386 SD870.9 g (min: 1270, max: 4300). The mean weight of the cases during surgery was 3636 SD452.7 g (min: 3000, max: 4130). The mean maternal age was 27.56 SD6.9 years (min: 20, max: 41). None of the cases had a dysmorphic appearance or findings suggestive of cardiac anomaly or genetic disorder.

**Fig. 1:**
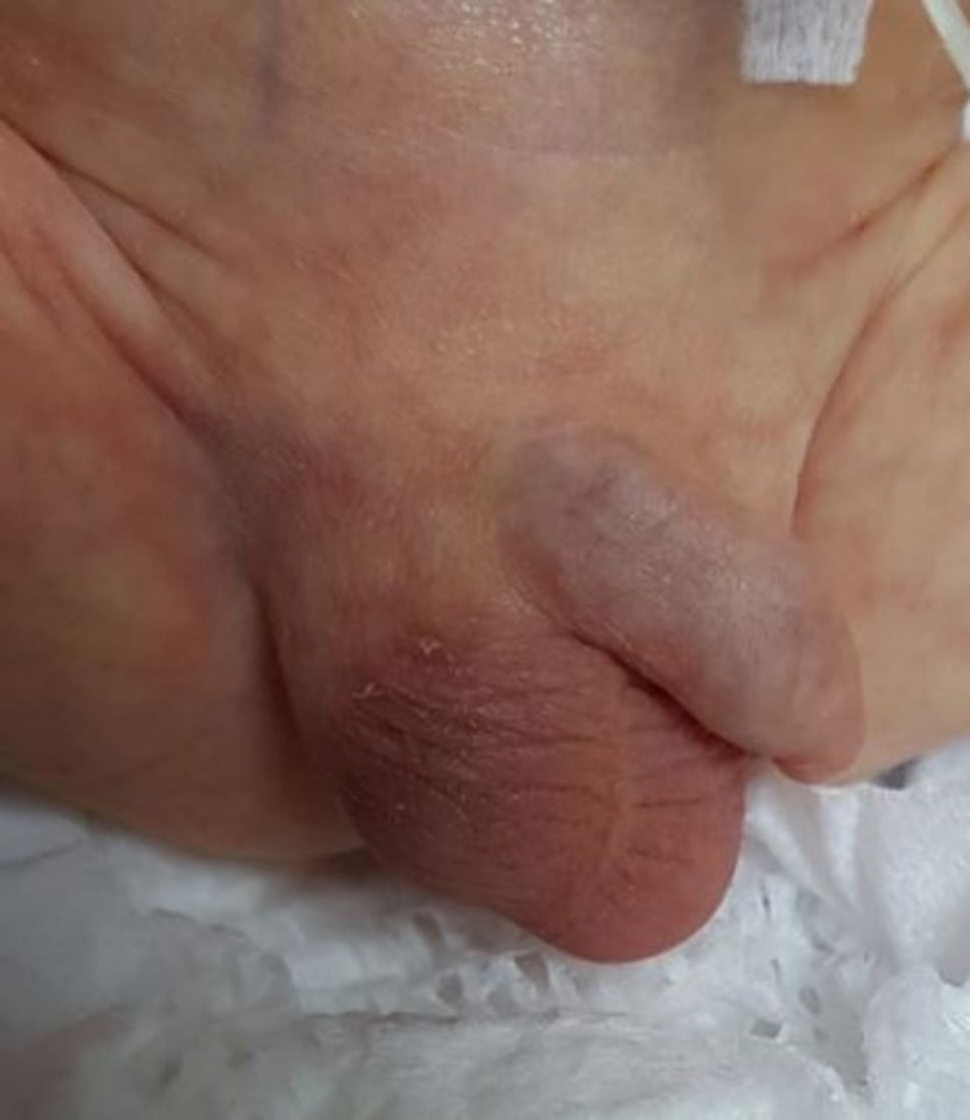
Right scrotal swelling, testicular mass formation, scrotal hyperemia and testicular elevation in newborn patient

Upon presentation to the pediatric surgery clinic, nine (52.9%) of the cases had complaints of scrotal swelling, four (23.5%) had hardness in the testicle, three (17.6%) had ecchymosis, two (11.8%) had redness on the scrotal skin, one (5.9%) had a testicular mass, and one (5.9%) had testicular enlargement. Three of them (17.6%) were referred by a neonatologist or pediatrician with the suspicion and initial diagnosis of ITT. Physical examination findings revealed testicular hardness in nine cases (52.9%), scrotal redness in six (35.3%), scrotal swelling in four (23.5%), testicular enlargement in four (23.5%), scrotal edema in four (23.5%), scrotal ecchymosis in four (23.5%), testicular fixation to the scrotal skin in three (17.6%), change in testicular position in three (17.6%), and testicular mass in two (11.8%). Figure 1 shows scrotal swelling, testicular mass formation, scrotal hyperemia and testicular elevation in a newborn patient (Fig. 1). Hydrocele was observed in the contralateral testicle in four (23.5%) cases. The torsion was on the left in ten cases (58.8%) and on the right in seven cases (41.2%). None of the cases had bilateral torsion.

**Fig. 2:**
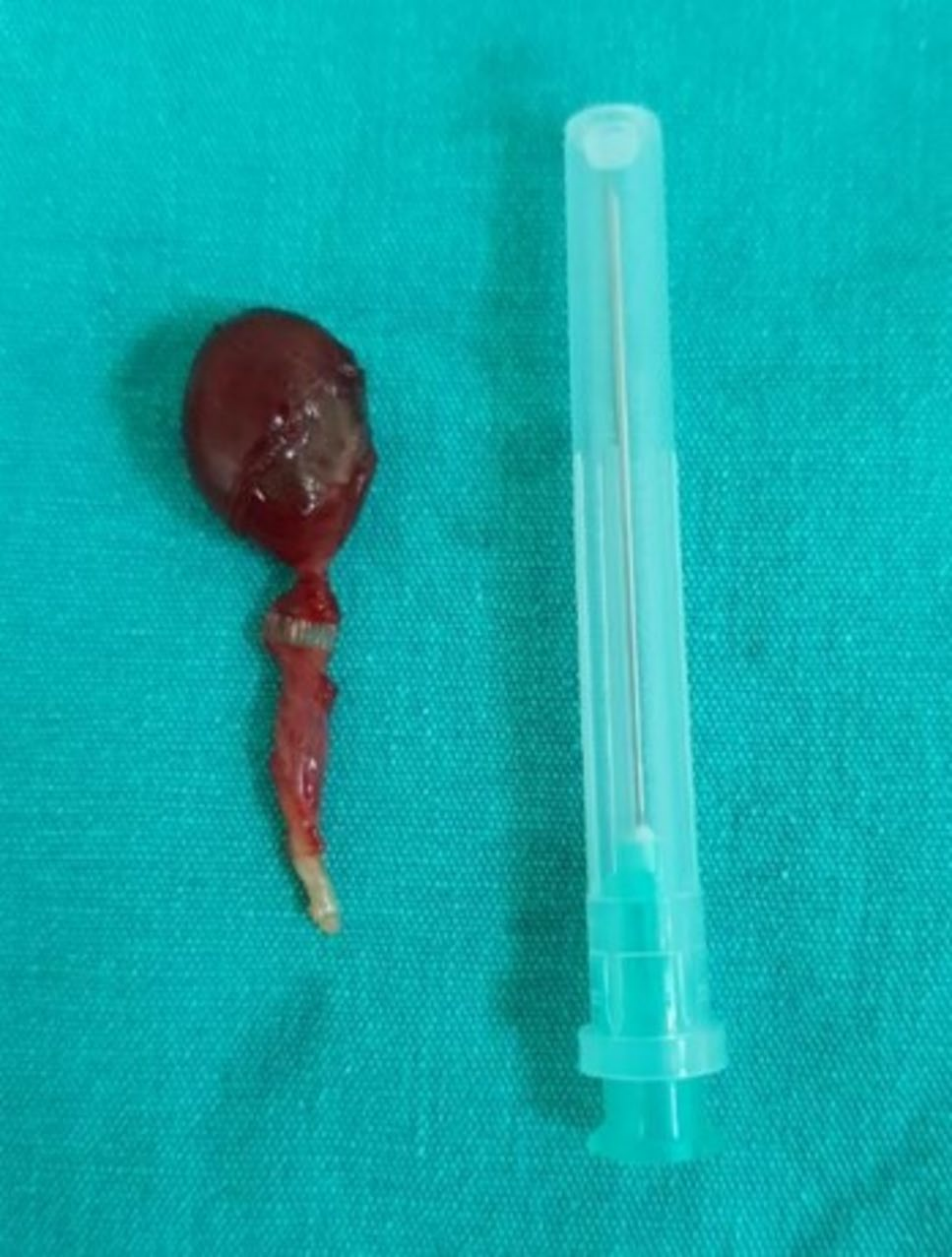
Orchiectomy material performed with high ligation

**Fig. 3:**
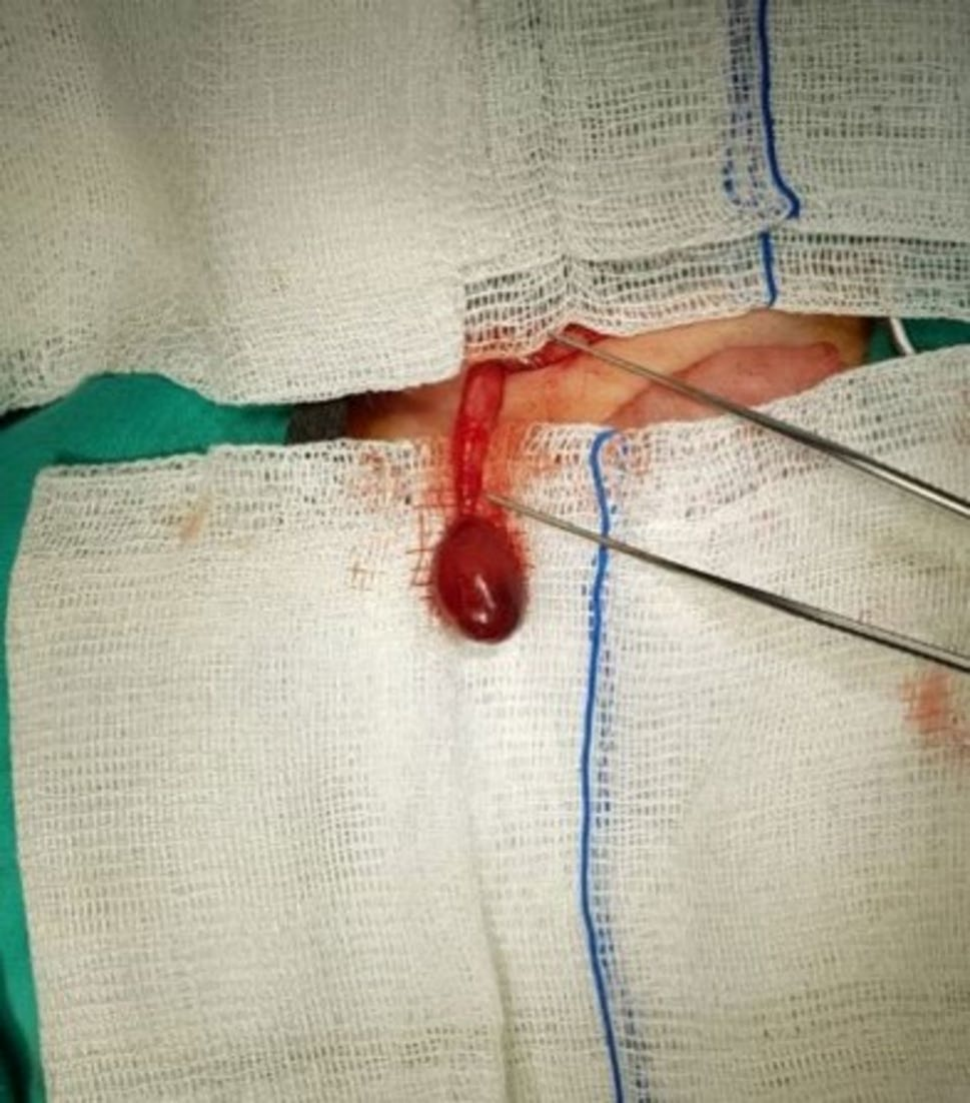
Extravaginal torsion of the testicle

Preoperative ultrasound and Doppler examination were performed in 10 cases (58.8%). None of the cases had testicular blood flow. Peritesticular fluid was noted in four cases (40%). Fourteen cases (82.4%) underwent orchiectomy, while three cases (17.6%) were managed with detorsion and orchiopexy. Figure 2 shows orchiectomy material performed with high ligation (Fig. 2). Additionally, prophylactic contralateral orchiopexy was performed in four (23.5%) cases, and undescended testicle surgery was performed in one (5.9%) case. Thirteen cases (76.5%) were explored via inguinal incision and four (23.5%) via a high scrotal incision. Of the nine (52.9%) recorded cases, the degree of torsion was 360° in three, 540° in one, 720° in three, 810° in one, and 1080° in one. The surgical records noted that nine cases (52.9%) had extravaginal torsion whereas three (17.6%) had intravaginal. Figure 3 shows extravaginal torsion of the testicle (Fig. 3).

The mean length of stay of the cases in the pediatric surgery clinic was 2.71 SD1.21 days (min: 1, max: 6). None of the cases had any complications during the surgery. Among the pathology examination results of the 14 cases who underwent orchiectomy, 12 were consistent with hemorrhagic infarction/necrosis, while germ cell aplasia and inflammatory fibromuscular-walled tissue fragments were reported in one case each. The testicles of all three cases managed with detorsion and orchiopexy were observed to be atrophic in follow-up ultrasonographic examinations.

## Discussion

ITT is a rare pathology, which has serious consequences such as testicular loss. There is still no consensus on how to treat and follow-up these cases. Although the etiology and predisposing factors of ITT are not fully elucidated, it is thought that increasing intrauterine pressure in the last trimester of pregnancy, and especially the pressure during normal spontaneous vaginal delivery, may trigger the cremaster reflex leading to torsion in cases where the tunica vaginalis and scrotum connection is weak [3]. Gestational diabetes, large gestational age (LGA), multiple pregnancies, breech arrival, and traumatic vaginal birth were proposed as risk factors for ITT [4, 5]. Likewise, four of our cases, one of which was the infant of a diabetic mother, were LGA. Interestingly, one case was premature, raising the question of whether prematurity is a predisposing factor for ITT, although it hasn’t been previously stated in the literature. Despite most studies reporting a higher incidence of ITT in vaginal births [6–8], the number of vaginal and Cesarean births was equal among our cases whose mode of delivery was known. Thus, the preferred delivery method in different countries should be considered when evaluating the relative contribution of vaginal birth as a risk factor of ITT.

It is generally believed that early diagnosis and intervention of ITT may increase the salvage rate of the testicles however, unfortunately, prenatal diagnosis is quite rare and often made incidentally during routine examinations. Hence, despite being an intrauterine event, ITT is usually diagnosed postnatally as demonstrated by a previous systematic review, in which only six of the 59 cases were reported to be diagnosed in utero [9]. In our study, only one case was diagnosed in the antenatal period at the 37th gestational week whereas the others were mostly (70.6%) diagnosed by physical examination and Doppler ultrasound on the first day after birth.

While Doppler ultrasound is a valuable tool in diagnosing ITT, it is mostly used only after suspicion is aroused by clinical findings. Furthermore, Doppler findings such as testicular enlargement, lack of blood flow, parenchymal heterogeneity, thickening of the tunica albuginea, and contralateral hydrocele may indicate testicular torsion [10, 11], yet are not unique for ITT [9]. Instead, the “whirlpool sign” was suggested as a pathognomonic ultrasound finding of torsion which has up to 96% diagnostic accuracy [12]. Preoperative ultrasound examination was performed in 11 cases in our study. The findings of all cases were consistent with the ultrasonographic findings of ITT reported in the literature and a whirlpool sign was detected at the hilus level in one case. The presence of physical examination findings such as swelling in the scrotum, testicular hardness, testicular enlargement, scrotal edema, mass, hyperemia, and ecchymosis without tenderness in all cases, in accordance with the literature, supported the Doppler ultrasound results for the diagnosis of ITT [3, 6, 13].

Despite previous studies claiming torsion to be more common on the left testicle [3], many studies argue against a notable side predilection [6, 7, 14]. In our study, although testicular torsion was more common on the left (58.8%), there was no significant difference between the two sides. All of our cases had unilateral torsion. In the literature, nearly all ITT cases are reported to be extravaginal. While extravaginal torsions (52.9%) were more common in our study as well, the rate of intravaginal torsion (17.6%) was well above the rates documented in most previous studies, such as a systematic review of 1336 cases in a total of 152 studies reporting 98.7% of ITT to be extravaginal [8].

Although there is not a universally accepted method for managing ITT, the generally preferred approach is the urgent removal of the necrotic testicle with orchiectomy and prophylactic contralateral orchiopexy [7, 15–17]. Additionally, in various studies, a minority of cases were managed with orchiopexy in hopes of salvaging the torsioned testicle, yet most of them eventually became atrophic during follow-up [6, 8]. Likewise, almost all of our cases were urgently operated on the first day after birth and 14 cases underwent orchiectomy, whereas detorsion and orchiopexy were performed in three cases after slight blood flow was observed in the explored testicle. Unfortunately, postoperative follow-up ultrasounds revealed that none remained viable. Unlike the literature, prophylactic contralateral orchiopexy was performed in only four (23.5%) cases to prevent risking injury to the healthy testicle which could possibly lead to anorchia.

## Conclusion

Similar to the literature, no testes could be saved in our study despite most cases being diagnosed and urgently operated on the day they were born [3, 4, 7]. Such low salvage rates create a dismal picture, raising questions regarding the efficacy of urgent surgical intervention in unilateral ITT cases. However, it may be overly simplistic to assume that all such cases are uniformly non-viable and it would not be unreasonable to think that the timing of torsion within the intrauterine period—early versus late—may influence testicular viability. Previous studies on postnatal torsion cases have shown that testes that were operated on in under 12 h were salvaged, yet it remains unclear whether a similar critical cutoff exists for intrauterine cases [18, 19]. This raises the question of whether defining a gestational age cutoff between early and late intrauterine torsion could help stratify prognosis and guide management strategies. More research is needed to determine whether, in select cases of suspected late intrauterine torsion, prompt diagnosis followed by expedited delivery could improve salvage rates. Collaboration with obstetricians and perinatologists and the use of fetal Doppler ultrasonography may facilitate timely detection. Rather than dismissing all intrauterine torsion cases, as unsalvageable, future studies should aim to differentiate between early and late intrauterine events to develop targeted, period-specific management approaches.

Since ITT is a rare pathology, studies on this subject are largely retrospective. One of the limitations of this study is that it is a single-centered case series analysis. Additionally, as the data was collected over a long period of time, the patient records were filled out by multiple people, making standardization difficult. Nonetheless, given the scarcity of research on this subject, we believe the results of this study collected from a rather large patient group over a 12-year period will contribute to the literature to better elucidate this pathology.

## Data Availability

The data that support the findings of this study are not publicly available due to their containing information that could compromise the privacy of research participants but are available from the corresponding author ODA via email upon reasonable request.
